# Evaluation of Combined Educational Methods on Motivational Interviewing for Final-Year Medical Students: Mixed Methods Study

**DOI:** 10.2196/89126

**Published:** 2026-04-29

**Authors:** Isaraporn Thepwongsa, Radhakrishnan Muthukumar, Pat Nonjui

**Affiliations:** 1 Department of Community, Family and Occupational Medicine, Faculty of Medicine, Khon Kaen University, Khon Kaen, Khon Kaen, Thailand; 2 Academic Affairs, Faculty of Medicine, Khon Kaen University, 123 Mittraparb Road, Muang Khon Kaen, Khon Kaen, 40002, Thailand, 66 43363588

**Keywords:** motivational interviewing, medical student, medical education, behavioral changes, combined educational methods, web-based learning, communication skills

## Abstract

**Background:**

Motivational interviewing (MI) is a patient-centered communication approach that supports health behavior change; yet, its integration into undergraduate medical curricula remains inconsistent. Combined learning models that comprise face-to-face instruction with structured web-based components may strengthen MI training, but evidence supporting their effectiveness among medical students, particularly in Asian contexts, is limited.

**Objective:**

This study evaluated the impact of a combined MI educational model on final-year medical students’ MI knowledge, confidence, and application in real patient encounters during clinical rotations.

**Methods:**

This study used a sequential explanatory mixed methods design. The quantitative component used a before-and-after study to evaluate changes in MI knowledge and confidence among final-year medical students enrolled in an Ambulatory Care course in 2024. All 130 students participated in a 2-hour interactive MI workshop, and 120 completed pre- and postintervention questionnaires assessing MI knowledge and self-reported confidence. Students were also provided access to a 3-hour web-based MI learning module, and learning-management system analytics were used to track engagement. The qualitative component consisted of semistructured interviews with 12 purposively selected students, conducted to explore their experiences applying MI during clinical encounters. Quantitative data were analyzed using paired-samples *t* tests, and qualitative data were analyzed using inductive conventional content analysis. Findings from both components were integrated during interpretation to provide a comprehensive understanding of the educational intervention.

**Results:**

Students demonstrated a significant improvement in MI knowledge following the educational intervention (pretest mean 8.87, SD 2.69; posttest mean 15.04, SD 2.99; *t*₁₁₉=–18.45; *P*<.001; η²=0.74). After the workshop, 96.9% (126/130) of students reported applying MI with patients, and 92.3% (n=120) agreed that the combined learning approach was adequate for supporting clinical use. Learning analytics data showed that 76.9% (n=100) of students enrolled in the web-based MI module, and 51% (n=51) completed all lessons. Students most frequently applied MI when counseling patients with diabetes, hypertension, and dyslipidemia, especially related to diet, physical activity, and medication adherence. Interview findings indicated that students mainly used brief MI, were most comfortable with engaging and focusing, and developed greater empathy, confidence, and patient-centered communication skills. Challenges included limited time during consultations, clinical workload, and difficulty applying all MI processes to complex cases.

**Conclusions:**

A combined MI learning approach integrating a short workshop with a web-based course was associated with higher MI knowledge scores and greater self-reported confidence among students, as well as reported use of MI-informed communication strategies during clinical encounters. Students perceived MI as a practical and ethically grounded communication approach that can enhance patient engagement, particularly in the management of chronic diseases. Introducing MI training longitudinally through a spiral curriculum, with opportunities for repeated practice and reinforcement, may help strengthen behavior-change communication competencies in undergraduate medical education.

## Introduction

### Background

Patient-centered communication is a fundamental component of effective clinical practice and plays a critical role in promoting health behavior change and improving clinical outcomes [1-5]. Physicians frequently engage in patient education during routine consultations, particularly when managing chronic diseases such as diabetes, hypertension, and cardiovascular conditions that require sustained lifestyle modification and self-management [1]. Evidence suggests that structured patient education interventions can improve clinical outcomes across a wide range of conditions, including cardiovascular disease, diabetes, respiratory disorders, and mental health conditions [1,2]. Effective patient education is typically supported by communication approaches that emphasize patient engagement, individualized counseling, and shared decision-making [6,7].

Among the various communication strategies used to support behavioral change, motivational interviewing (MI) has emerged as one of the most widely studied and evidence-based approaches [8]. MI is a collaborative, patient-centered counseling method developed by Miller and Rollnick [8] that aims to strengthen individuals’ motivation and commitment to change by exploring and resolving ambivalence [9]. The approach emphasizes empathy, collaboration, and respect for patient autonomy while guiding patients toward identifying their own motivations for change. Over the past 3 decades, MI has been applied across a wide range of health care settings and clinical conditions [9,10].

A growing body of evidence demonstrates that MI can effectively support improvements in health behaviors and clinical outcomes [9-15]. Systematic reviews and meta-analyses have shown that MI interventions are associated with increased physical activity, smoking cessation, weight reduction, improved glycemic control, and reductions in alcohol consumption and other health-risk behaviors [11-14]. MI has also been successfully implemented in primary care settings, where physicians frequently counsel patients on lifestyle modification and chronic disease management [15-17]. However, despite its demonstrated effectiveness, studies indicate that motivational and patient-centered communication techniques remain underused in routine clinical practice, partly due to insufficient training among health care professionals [16,17].

Recognizing the importance of behavior-change communication, medical education programs have increasingly explored ways to incorporate MI training into undergraduate curricula [1]. Previous studies have shown that MI can be successfully taught to medical students and other health professional trainees using a variety of instructional strategies, including workshops, role-play exercises, coaching, and supervised practice [18,19]. These educational interventions have been associated with improvements in students’ communication skills, confidence, and readiness to support patient behavioral change [20-22]. In addition, training in MI may help students develop transferable clinical communication skills such as reflective listening, open-ended questioning, and affirmation, which are valuable across many areas of clinical practice [6,23].

Despite these promising findings, several challenges remain in integrating MI training into medical curricula. Existing educational programs vary widely in duration, teaching methods, and assessment approaches, and there is no widely accepted standard model for MI education in undergraduate medical training [1,18,19,24-26]. In many programs, MI instruction is limited to short workshops without sufficient opportunities for reinforcement, practice, or longitudinal integration. As a result, the sustained application of MI skills in clinical practice remains inconsistent [10,24,25].

Multifaceted or combined educational methods comprising face-to-face instruction with web-based learning modules have been proposed as a promising strategy for improving MI training [14,27]. Such approaches allow learners to acquire foundational knowledge through flexible online learning while using in-person sessions for interactive practice and feedback. Emerging evidence suggests that combined educational methods can enhance learner engagement, knowledge retention, and skill development compared with traditional teaching methods alone [18,19,28]. However, relatively few studies have examined the effectiveness of combined MI training models among undergraduate medical students, particularly in Asian and low- and middle-income educational contexts [18,19,26].

Although MI training has been studied in medical education, relatively little research has examined how combined educational approaches comprising in-person workshops with web-based modules can be implemented within undergraduate medical curricula in resource-constrained educational settings. This study therefore evaluated the implementation of a combined learning approach for introducing MI to final-year medical students in Thailand and explored students’ learning experiences and reported application of MI during clinical training.

### Our Medical School Curriculum

Our medical school, located in northeastern Thailand, enrolls approximately 310 students annually. The 6-year undergraduate medical curriculum consists of a premedical phase, a preclinical phase, and a clinical phase, totaling 259 academic credits [29,30]. The first year focuses on premedical education (38.5 credits), while the second and third years emphasize preclinical sciences (76.5 credits). Clinical education occurs during years 4 to 6 and comprises 96 credits during years 4 and 5, followed by 48 credits during the final-year clinical rotations [30].

The learning outcomes of the curriculum are categorized into 3 domains: fundamental knowledge, professional competency, and professionalism. Communication skills are included as a core competency under the professionalism domain. Communication training is integrated throughout both preclinical and clinical courses; however, structured training specifically focused on behavior-change communication techniques has not been mandatory in the curriculum.

Before the 2024 academic year, some students may have been exposed to MI informally during clinical rotations in psychiatry. Beginning in the 2024 academic year, MI training was formally introduced as a compulsory component of the final-year curriculum within the Ambulatory Care in Primary Care Setting course, where this study was conducted.

Final-year medical students participating in this course have already completed core clinical rotations and are actively involved in outpatient care under faculty supervision. During these rotations, students conduct patient interviews, participate in clinical decision-making, and provide counseling for chronic disease management. This clinical environment provides opportunities for students to apply behavior-change communication techniques such as MI during real patient encounters.

## Methods

### Study Design

#### Overview

This study used a sequential explanatory mixed methods design consisting of 2 complementary phases. In the first phase, a quantitative before-and-after evaluation was conducted to assess changes in final-year medical students’ knowledge in MI following participation in the educational intervention.

In the second phase, semistructured qualitative interviews were conducted after completion of the quantitative data collection to further explore students’ experiences in learning and applying MI during clinical encounters. The qualitative component was designed to help explain and contextualize the quantitative findings, particularly regarding how students applied MI in real clinical settings and the challenges they encountered when using MI with patients. The integration of quantitative and qualitative findings provided contextual insights into students’ learning experiences and complemented the quantitative results.

As the training was implemented as part of the required curriculum for all students, a comparison group was not feasible; therefore, this study was designed as an educational program evaluation rather than an experimental effectiveness study.

#### Participants

This study’s population consisted of final-year medical students enrolled in the “Ambulatory Care in Primary Care Setting” course during the 2024 academic year (May 2024 to April 2025). This course is a required component of the clinical curriculum and includes outpatient training in primary care settings.

Students who participated in the pilot testing of the questionnaire instruments (n=28) were excluded from this study to avoid potential bias related to prior exposure to the survey tools.

Sample size estimation was conducted using G*Power (version 3.1.9.7; Heinrich-Heine-Universität Düsseldorf) for paired comparisons of pre- and posttest scores [31]. Assuming a moderate effect size of 0.5, a statistical power of 80%, and a significance level of 0.05, the minimum required sample size was estimated to be approximately 50 participants.

The assumed effect size of 0.5 was selected based on conventions for moderate effect sizes commonly used in educational intervention studies and prior research evaluating communication skills and MI training among health care trainees.

Although all eligible final-year medical students enrolled in the course were invited to participate in this study, the power analysis was conducted before data collection to verify that the available cohort size would be sufficient to detect meaningful differences in knowledge scores between pre- and posttests. As the available cohort included 130 students, the sample size exceeded the minimum requirement estimated by the power analysis.

### Educational Interventions

The educational intervention was designed to introduce foundational principles of MI and to provide opportunities for students to apply MI concepts in clinical contexts. The intervention combined a face-to-face interactive workshop with a structured web-based learning module and brief MI guides, allowing students to acquire theoretical knowledge and practice communication strategies relevant to behavior-change counseling in primary care.

### Workshop

A 2-hour interactive workshop on MI was provided. The students attended the workshop in small rotation groups (25‐28 students per group; 6 rotations throughout the academic year). The workshop facilitators (IT and PN) are faculty members in family medicine and medical education with experience in behavioral counseling in primary care. They have completed formal MI training programs and are continuing professional development activities related to MI.

The workshop content included an overview of health-behavior change theory, the principles and spirit of MI, and the 4 core MI processes (engaging, focusing, evoking, and planning). Students were introduced to the use of key MI communication strategies, including open-ended questions, affirmations, reflective listening, and summaries.

Interactive learning activities were incorporated to support skill development. These included case-based discussions, role-play exercises, and guided demonstrations of brief MI techniques. Students practiced applying MI strategies to simulated clinical scenarios commonly encountered in primary care, particularly in counseling patients with chronic conditions such as diabetes and hypertension.

### Web-Based Course

In addition to the workshop, students were provided access to a structured web-based MI learning module designed to reinforce key concepts and support self-directed learning. The module contained approximately 3 hours of learning material and included short lessons covering MI principles, stages of change, and practical applications in patient consultations.

The online course incorporated interactive elements such as quizzes, self-reflection exercises, and case-based learning activities to encourage active engagement. The web-based curriculum was developed following established principles for effective online education [32]. The web-based module was provided as supplementary learning material to reinforce the workshop content. Students could voluntarily access and complete the course at their own pace through the medical school’s learning management system.

Completion of the web-based module was defined according to the platform’s predefined criteria, which required students to complete all lessons and associated activities in the online course.

### Brief MI Guide

A brief MI guide was provided as a downloadable resource within the web-based course and distributed during the workshop. The guide summarized key MI communication principles and included practical examples of open-ended questions, reflective statements, and strategies for exploring patients’ motivation for behavior change. It also provided practical prompts to help students incorporate MI-informed communication into routine outpatient consultations. The guide served as a quick reference tool during clinical training rather than a comprehensive MI training manual. The resource was adapted from the “A Taste of MI” exercise included in the MI foundational training developed by Miller, Rollnick, and Moyers [33] and has been described in our previous publication [27].

The learning objectives of the intervention were to (1) introduce students to the principles and spirit of MI; (2) familiarize students with the four MI processes—engaging, focusing, evoking, and planning—and with fundamental MI communication skills, including open-ended questions, affirmations, reflective listening, and summaries; and (3) support the initial application of MI-informed communication strategies, including brief MI techniques, during clinical encounters.

Given the limited duration of the training, the program was designed to provide introductory exposure to MI concepts and basic communication strategies rather than to develop full MI proficiency.

The interactive workshop was a required component of the Ambulatory Care course, and therefore, all enrolled students attended the workshop session. In contrast, the web-based MI learning module was provided as a supplementary self-directed learning resource, allowing students to access and complete the lessons voluntarily at their own pace through the learning management system.

### Quantitative Component

A before-and-after design was applied to assess students’ knowledge before and after participation in the MI training. Students completed the pretest questionnaire before participating in the MI workshop. The baseline questionnaire included demographic characteristics (age and sex) as well as information about students’ prior exposure to MI and other communication strategies used in patient counseling. Additional items assessed whether students had previously received training related to MI and their experiences with behavior-change communication during clinical rotations. These baseline variables were collected to characterize this study’s population and to provide contextual information regarding students’ prior familiarity with MI and communication skills before participating in the educational intervention. The posttest questionnaire was administered immediately after completion of the workshop to assess immediate changes in knowledge and confidence following the instructional session. The web-based module was provided as supplementary learning material that students could access voluntarily after the workshop to reinforce MI concepts during their clinical training. The instruments were developed by the research team (IT and RM) based on the relevant literature [14] and reviewed by experts in medical education and general practice to ensure content validity. Three independent reviewers assessed each item for clarity, relevance, and necessity. The questionnaires included multiple-choice, yes or no, open-ended, and Likert-scale questions evaluating knowledge, attitudes, and self-reported application toward MI.

Knowledge scores were calculated by summing the number of correct responses to the multiple-choice questions, generating a total knowledge score for each participant in both the pre- and posttests.

For attitudinal measures, the postintervention questionnaire included items assessing students’ self-reported confidence in applying MI during patient consultations. Confidence was assessed across the 4 MI processes—engaging, focusing, evoking, and planning. Participants rated their confidence using a 5-point Likert scale ranging from 1 (not at all confident) to 5 (very confident). Students also reported the extent to which they applied MI during clinical encounters using a 5-point Likert scale ranging from 1 (used the least) to 5 (used the most). The questionnaire items were developed based on the MI framework described by Miller and Rollnick and were reviewed by experts in medical education and general practice to ensure content validity. After piloting with 28 students, minor revisions were made. Internal consistency reliability was assessed for the Likert-scale items measuring confidence related to MI, yielding a Cronbach α coefficient of 0.93. The survey instrument used in this study is available from the corresponding author upon reasonable request.

### Qualitative Component

Semistructured interviews were conducted with students who had completed both the pre- and posttests. The semistructured interview guide was developed by the research team based on this study’s objectives, findings from the quantitative phase, and relevant literature on MI education and communication skills training in medical education. The interview questions explored several key domains, including students’ experiences applying MI in clinical encounters, which MI processes or techniques they used most frequently, perceived changes in their communication style following the training, challenges encountered when attempting to apply MI during patient consultations, perceived benefits of MI for patient communication, and students’ perspectives on the most appropriate timing and methods for teaching MI in the medical curriculum. All interview participants were drawn from the same cohort of final-year medical students who participated in the quantitative component of this study.

Students were informed about the qualitative component after completing the MI training and posttest questionnaire and were invited to participate in voluntary interviews. Students who expressed interest were contacted by the research team and scheduled for an interview.

A total of 12 students were selected using purposive sampling to ensure representation from different periods of course participation during the academic year. Specifically, students were selected from early, middle, and late course rotations in order to capture a range of experiences with learning and applying MI during clinical training.

The sample size was guided by the principle of thematic sufficiency commonly used in qualitative research exploring participant experiences. During analysis, the research team observed that key themes began to recur across interviews, suggesting that sufficient depth of information had been obtained for the purposes of this exploratory qualitative component.

Interviews were conducted in May 2025 via Google Meet and lasted approximately 15‐20 minutes. The relatively brief duration reflected scheduling constraints during students’ clinical rotations; however, the semistructured format enabled focused discussion of key topics related to students’ experiences with learning and applying MI. All interviews were audio-recorded with participants’ consent, transcribed verbatim, and returned to participants for verification to ensure transcription accuracy.

The semistructured interview guide used in this study is available from the corresponding author upon reasonable request.

### Data Analysis

#### Quantitative Analysis

Statistical analyses were performed using IBM SPSS Statistics for Windows (version 20.0; IBM Corp). Paired-samples *t* tests were used to compare the mean knowledge scores before and after the intervention. Analyses were conducted using pairwise deletion, meaning that only participants who had both pre- and posttest responses for the relevant variable were included in the paired comparisons. Participants with missing responses in either the pretest or posttest were excluded from the paired analysis for that variable.

These measures were designed to assess students’ perceived knowledge, confidence, and self-reported use of MI-informed communication strategies, rather than to provide an objective assessment of MI competency.

#### Qualitative Analysis

The interview data were analyzed using conventional content analysis, following the methodological approach described by Hsieh and Shannon [34]. An inductive approach was adopted, involving repeated reading of transcripts to identify initial codes, which were subsequently grouped into broader categories and themes. Initial coding was conducted by 1 researcher (IT), after which emerging codes, categories, and interpretations were reviewed and discussed with the research team to enhance analytical rigor and credibility.

#### Integration of Quantitative and Qualitative Data

The 2 data strands were integrated during interpretation to provide a comprehensive understanding of the intervention’s impact. Quantitative findings demonstrated measurable improvements in knowledge and confidence, while qualitative insights illustrated how students experienced, applied, and reflected on MI in clinical contexts. This integration allowed qualitative findings to complement and provide contextual insights into the quantitative results, strengthening the overall interpretation of this study’s findings.

#### Ethical Considerations

This study was approved by the Khon Kaen University Human Research Ethics Committee (project number HE631031). Participation was voluntary, and all students were informed of their rights to withdraw at any stage without academic consequences. Informed consent was obtained from all participants before data collection. Confidentiality and anonymity were maintained throughout the research process. Participants were encouraged to share honest feedback, as their insights were considered valuable for improving educational approaches in behavioral change communication training. Participation was voluntary, and no incentives were provided.

## Results

### Overview

Table 1 shows demographic data of the participating final-year medical students. None of the participants had prior MI training or course completion within the past 3 years. Table 2 shows student participation in components of the MI training program. All 130 final-year medical students attended the required 2-hour MI workshop as part of the Ambulatory Care course. The web-based MI learning module was offered as a voluntary supplementary learning resource. Learning-management system analytics showed that 100 (76.9%) students accessed or enrolled in the online module. Among those who accessed the module, 51 (51%) students completed all required lessons and activities according to the platform’s completion criteria. Across students who accessed the module, the mean number of lessons completed was 9 of 19 lessons, indicating varying levels of engagement with the online content. Participants’ enrollment time in the course ranged from 0 to 481 minutes, with a mean of 71.98 (SD 92.49). The number of completed lessons by participants ranged from 0 to 19 lessons, with a mean of 9.19 (SD 8.87). These findings indicate variable engagement with the web-based module, reflecting the self-directed nature of the online component.

**Table 1.** Demographic data of the participating final-year medical students.

Demographic data	Values, n (%)
Sex (n=128)	
Males	69 (53.9)
Females	59 (46.1)
Age (year; n=127)	
Mean (SD)	23.58 (0.61)
Range	22-25
Prior exposure to MI^a^ within the past 3 years (n=127)	
Yes	18 (14.2)
No	109 (85.8)
Prior MI training or course completion within the past 3 years (n=127)	
Yes	0 (0.0)
No	127 (100.0)
Based on your experiences, which techniques did you use to help patients change their behaviors (n=127)	
Giving information	120 (94.5)
Advising	112 (88.2)
Order or instruct to do	12 (9.4)
Motivating	65 (51.2)
Never	3 (2.4)

a MI: motivational interviewing.

**Table 2.** Student participation in components of the MI^a^ training program.

Training component	Values
Attended MI workshop (required), n (%)	130 (100)
Accessed or enrolled in web-based module, n (%)	100 (76.9)
Completed all web-based lessons, n/N	51/130
Lessons completed (among enrolled students; N=19), mean (SD)	9 (8.87)

a MI: motivational interviewing.

Most final-year medical students expressed strong needs and expectations regarding MI. Before completing the MI workshops, the majority wanted to strengthen their skills in choosing words carefully, applying stages of change, motivating patients, and enhancing communication for health-behavioral change (Table 3). Their learning expectations were consistently high, with mean scores near 4 on a 5-point scale, particularly for MI skills, knowledge, and helping patients control their diseases through MI (Table 4). Regarding specific behavioral areas, students prioritized skills related to oral medication adherence, diet and nutrition, doctor appointment adherence, and injection drug administration (Table 5). Overall, the data highlight the strong motivation among students to develop practical MI communication and counseling competencies that directly support patient self-management and lifestyle modification.

**Table 3.** Skills that participants wanted to develop to help patients with diabetes, other chronic diseases, or the general population in modifying health behaviors (before completion of the MI^a^ workshops).

Skills that participants wanted to improve (n=127)	Values, n (%)
Choosing words carefully to enhance motivation	96 (75.6)
Applying the stage of change	95 (74.8)
Motivating	90 (70.9)
Communication skills to enhance motivation	90 (70.9)
Counseling	84 (66.1)
Facilitating successful health-behavioral change in patients	84 (66.1)

a MI: motivational interviewing.

**Table 4.** Participating final-year medical students’ expectations for learning the MI^a^ course.

Expectation in learning on MI (n=127)	Values, mean (SD)^b^
MI skills	4.06 (0.84)
Control patients’ illnesses or diseases after applying MI with them	3.99 (0.72)
MI knowledge	3.97 (0.80)
Patients can change their behaviors after applying MI with them	3.94 (0.77)
Prompt use for MI	3.91 (0.87)
Teaching colleagues about MI	3.62 (0.91)

a MI: motivational interviewing.

b Mean was calculated using a 5-point Likert scale ranging from 1 (not at all expected), 2 (slightly expected), 3 (moderately expected), 4 (very much expected), and 5 (fully expected).

**Table 5.** Specific behaviors that the participating final-year medical students needed skills and knowledge in helping patients change.

Specific behaviors	Values, mean (SD)^a^
Oral medication administration	4.24 (0.79)
Diet and nutrition	4.20 (0.76)
Doctor appointment adherence	4.20 (0.83)
Injection drug administration	4.15 (0.84)
Exercise	4.13 (0.80)
Counseling skills for behavioral change	4.09 (0.75)
Behavioral changes in all aspects	4.06 (0.83)
Updating knowledge and skills in patient care	4.05 (0.83)
Working with the patient’s family	3.94 (0.82)

a Mean was calculated using a 5-point Likert scale ranging from 1 (least needed), 2 (slightly needed), 3 (moderately needed), 4 (very needed), and 5 (most needed).

After attending the MI workshop, 126 of 130 (96.9%) final-year medical students reported attempting to apply elements of MI during patient encounters within the preceding 2 weeks. Most participants (n=120, 92.3%) indicated that the combination of a 2-hour workshop and web-based learning was sufficient for applying MI in clinical practice. Students perceived MI as a useful communication approach for supporting health-behavioral change (mean 4.47 of 5, SD 0.67).

After completing the MI workshop, the final-year medical students who applied MI techniques in real clinical settings predominantly used MI to support patients in improving their diet, physical activity, and medication adherence (Table 6), particularly among those with diabetes, hypertension, and dyslipidemia (Table 7). Students reported applying elements of MI during patient encounters and demonstrated moderate to high confidence in applying MI across all 4 MI processes (Table 8). The highest confidence score was observed for engaging (mean 3.73, SD 0.78), followed by focusing (mean 3.65, SD 0.74), planning (mean 3.52, SD 0.87), and evoking (mean 3.38, SD 0.90). These findings suggest that students felt relatively comfortable initiating and structuring MI conversations but reported slightly lower confidence in more advanced MI processes such as evoking change talk.

**Table 6.** Issues that the participating final-year medical student applied MI^a^ to the patients.

Issues that the students applied MI with the patients (n=126)	Values, n (%)
Diet change	117 (92.9)
Exercise	100 (79.4)
Medication intake	67 (53.2)
Quitting alcohol consumption	43 (34.1)
Decision-making about treatment	42 (33.3)
Smoking cessation	39 (31)
Doctor appointment adherence	37 (29.4)

a MI: motivational interviewing.

**Table 7.** Types of patients with whom the final-year medical student applied MI^a^.

Type of patients (n=126)	Values, n (%)
Diabetes	119 (94.4)
Hypertension	112 (88.9)
Dyslipidemia	111 (88.1)
Obesity	47 (37.3)
Stroke	12 (9.5)
Cardiovascular disease	8 (6.3)

a MI: motivational interviewing.

**Table 8.** MI^a^ processes that were used by the final-year medical student to help patients change their behaviors and their level of confidence to use each MI process.

MI process (n=126)	Level of use, mean (SD)^b^	Level of confidence, mean (SD)^c^
Engaging	3.95 (0.93)	3.73 (0.78)
Focusing	3.59 (0.93)	3.65 (0.74)
Evoking	3.47 (0.97)	3.38 (0.90)
Planning	3.48 (1.10)	3.52 (0.87)

a MI: motivational interviewing.

b Mean was calculated using a 5-point Likert scale ranging from 1 (used the least), 2 (used a little), 3 (used moderately), 4 (used a lot), and 5 (used the most).

c Mean was calculated using a 5-point Likert scale ranging from 1 (not at all confident), 2 (somewhat not confident), 3 (neutral), 4 (confident), and 5 (very confident).

### Comparing Participating Medical Students’ Knowledge Scores Before and After Workshop Completion

A total of 130 final-year medical students participated in the MI workshop. Of these, 126 (96.9%) students completed the pretest questionnaire, and 120 (87.5%) students completed the posttest questionnaire. Students who did not complete the posttest were primarily absent during the postintervention data collection session or did not submit the questionnaire. Paired analyses were conducted using data from the 120 students who completed both the pre- and posttest. The proportion of missing data for the paired analysis was therefore small and unlikely to substantially affect the results. A paired-sample *t* test was conducted to examine differences in knowledge scores before and after participation in the MI training. There was a statistically significant difference in the knowledge test scores from time 1 (mean 8.87, SD 2.69) to time 2 (mean 15.04, SD 2.99), *t*_119_=−18.45, *P*<.001 (2-tailed). The mean increase in knowledge test score was −6.18 (SD 3.66), 95% CI −6.84 to −5.51. The eta-squared statistic indicated a large effect size (eta-squared=0.74).

### Insights From Participant Interviews

A total of 12 final-year medical students voluntarily participated in the semistructured interviews, representing 3 groups categorized by their enrollment period in the MI course: students who completed the course at the beginning of the 2024 academic year (May to August 2024), coded as group A; those who completed it in the middle of the academic year (September to December 2024), coded as group B; and those who completed it at the end of the academic year (January to April 2025), coded as group C. To maintain participant confidentiality while distinguishing individual responses, each quotation is identified using a code consisting of a letter and a number. The letter (A, B, or C) represents the interview group based on the period of course participation, and the number represents the individual participant within that group.

The qualitative analysis identified several themes related to students’ experiences learning and applying MI during clinical training, including experiences using MI in clinical encounters, changes in communication style, barriers to implementation, perceived advantages and benefits of MI, development of confidence, and students’ perspectives on MI education.

According to the semistructured interviews (Table 9), the final-year medical students reported applying MI primarily in brief forms during patient encounters, particularly with patients managing chronic conditions such as diabetes, hypertension, dyslipidemia, and gout. They identified engaging as the most frequently used and comfortable skill, whereas evoking and planning were used less often due to time limitations and lower confidence. After completing the MI course, the students observed noticeable improvements in their communication, becoming more empathetic, reflective, and patient-centered. They perceived MI as a valuable approach that enhanced patient motivation, strengthened doctor-patient relationships, and promoted ethical and collaborative care. Nonetheless, challenges such as heavy workloads, time constraints, and communication barriers with some patients were reported. Most students expressed increased confidence in using MI and preferred learning it during their fifth year, when clinical exposure allowed sufficient opportunity for practice before the externship year. They also emphasized that effective MI instruction should integrate lectures with practical components, such as role-play, video demonstrations, and feedback sessions, ideally delivered through blended or flipped classroom models, to reinforce both theoretical and applied communication skills.

**Table 9.** The participating final-year medical students’ experiences in and perceptions toward the use of MI^a^ with patients.

Final-year medical students’ experiences in and perceptions toward the use of brief MI with patients	Comments
Theme 1: students’ experiences applying MI in clinical encounters Final-year medical students reported applying MI during patient encounters, most often in the form of brief MI rather than the full 4-process model (A1-A4, B1-B5, and C1-C3). They most commonly used MI with patients managing NCDs^b^ such as diabetes, hypertension, dyslipidemia, or gout (B1, A1, B3, C1, and C3), and in some cases to support smoking cessation (C3) or alcohol reduction (B2). Across interviews, students identified engaging as the MI skill they used most frequently and felt most comfortable with, while evoking and planning were used less often because of limited time, clinical workload, or lower confidence in applying the full sequence (A1, B2, and B4). Many students explained that although they could not always perform all MI processes, they consistently incorporated key elements such as open-ended questioning, reflection, and affirmation into routine consultations (B5 and C3).	“I used MI with patients with diabetes, hypertension, dyslipidemia and gout.” (B1) “In general, I try to explore the root cause of the patient’s problem. I haven’t had the opportunity to use the full MI process yet, but I try to engage them in talking about what happened and what consequences have occurred and tried brief MI with all patients with diabetes.” (B2) “I use every MI process and apply brief MI with all patients, mainly focusing on engaging. Sometimes patients ask, ‘Will I get to see your supervisor?’ or say things to make me feel like I’m not trustworthy enough, and insist that they would rather talk with my supervisor.” (B4) “Engaging is the basic process and the easiest to use. Affirmation is important for maintaining positive communication. Open-ended questions have some limitations because patients may not understand us, making them harder to use. I had already used some of these techniques before knowing MI through this course, and during the training, I felt good realizing that I had been using them already.” (B5) “Engaging is used very often, especially when adjusting medication for diabetic patients. I ask questions like, ‘Why is the control not achieved?’ or ‘Is there anything that can be changed?’—but I haven’t gone as far as the evoking process yet for the full MI but I tried to used brief MI. I also use the concept of the stages of change to assess which stage the patient is in and whether they are ready to change their behavior.” (A1) “I use Engaging in the outpatient department because we usually focus only on the patient’s presenting problem. In reality, we can inspire or spark awareness in the patient. For example, a patient came with diabetes and also smoked. At first, I didn’t focus on the smoking issue, but I tried to use reflection, which helped the patient realize that the number of cigarettes he smoked was quite high. When someone reflected this back to him, the patient said, ‘I know it’s a lot, right, doctor?’ In that case, I went through all four MI processes. It felt like small talk that helps initiate the conversation.” (C3)
Theme 2: changes in students’ communication style after MI training Final-year medical students reported clear changes in their communication style after completing the MI course, most commonly integrating engaging, open-ended questioning, and affirmation into their routine consultations (B1, B2, B4, and C1). Engaging was the MI process used most frequently, often combined with focusing to help patients better understand their condition and explore their own reasons for behavioral change (B4 and C1). Several students also described increased use of reflection and empathic listening, which helped patients express their motivations more freely and led to deeper conversations (C3). Although evoking and planning were used less consistently, students reported feeling more confident overall and more capable of supporting patient-led change, moving away from directive advice-giving toward a more collaborative style of consultation (A1 and C3).	“Used Engaging and tried to ask open-ended questions. Changed my way of working significantly after learning MI. After the course, I felt more confident using it and even tried applying it with my family members.” (B1) "After learning about Motivational Interviewing, what I’ve continued to use is open-ended questions. I ask open-ended questions to let them share, and then I practice empathic listening. Before the course, I had used affirmations occasionally, but after the course, I began using them more often. Giving affirmations helps patients feel proud of themselves and more motivated.” (B2) “The skill I continue to use regularly is engaging. I even applied it with my grandfather at home regarding his smoking. We had conversations using engaging and focusing, and as a result, he reduced his smoking. Affirmation is the skill I use the most.” (B4) “Engaging is probably the skill that has stayed with me after the course because I don’t usually see patients long term. Most of the time, I see them for just one visit. So, I use engaging and focusing—patients don’t always understand what they have, but we help them understand themselves better and improve their compliance.” (C1) “I use reflection more often now. At first, I would just keep asking questions and taking notes based on what the patient said. But when I started using reflection, it made me pause and think, and it also helped the patient reflect on themselves at the same time. As for affirmation, it is very important. Positive reinforcement has always worked well. What the patient gains is the feeling that someone cares and is interested in them, which helps them open up more. This makes it easier when it comes time to offer advice.” (C3) “Before and after the course, I think there’s a big difference. I had never really helped patients change their behavior, but in real life, it’s very important. If we can help them reach a stage where they’re ready to change, it has a significant impact on the condition they are dealing with.” (A1)
Theme 3: challenges and barriers to using MI in clinical practice Final-year medical students reported several challenges when attempting to use MI in clinical settings. A major barrier was time pressure and high patient load, which made it difficult to perform all MI processes and often resulted in reverting to traditional directive advice-giving (B3, A2, and B5). Many students also noted that their limited experience and developing confidence in MI techniques led to hesitation, disrupted communication flow, or incomplete use of the approach (B1). Students described difficulties in choosing the right wording, keeping conversations focused, and managing open-ended questions—which sometimes led patients off-topic and required more time than was available (A3 and C2). Additional obstacles included challenges communicating with older adult patients who had hearing problems or preferred to speak only with a doctor rather than a student (B4). Some students also encountered patients with fixed beliefs or preconceived online information, which made behavioral change difficult unless MI skills were used fluently and confidently (C1).	“While using it, I couldn’t think of how to proceed, so the interaction wasn’t smooth. I’ve only just started using it and I’m not yet proficient. My skill and experience are still limited.” (B1) “How to use words so they understand us. Choosing the right words/change talks is challenging. If I could follow up with the same patient, I think I would be able to use this effectively—it would be doable if it’s the same person. In real life, it takes a lot of time to listen. I believe it can be applied to everyone, but for now, I haven’t used it with every patient.” (C2) “With open-ended questions, patients often talk about many things off-topic, which takes a lot of time.” (A3) “Because of the time constraints and the high number of patients, there’s very little time left for each consultation. If I try to use MI, it becomes impractical—I won’t be able to finish on time, and it makes other patients wait longer.” (B3) “Elderly patients often have trouble hearing, so I have to speak loudly, and they are usually not very cooperative. It takes a long time to talk with them. Patients prefer to make plans with the doctor (not with the student).” (B4) “Because of limited time in real-life practice, I may not be able to use every step—only the basic ones. Patients may not come for counseling but rather for treatment. Some principles may not be fully understood at first, until I gain more experience using them with patients.” (B5) “If we are unable or lack the necessary skills, we may not be able to apply the principles in practice. Many patients nowadays have access to technology. They search for information on their own and come with fixed ideas or a fixed mindset. If we don’t have the skills, we won’t be able to help them.” (C1) “Conversations take time, and because of the large number of patients, we are unable to complete the full process. The time we have is limited, so we end up giving direct advice in the usual way—such as about alcohol or diet—which fits within the time limit, but the patients still don’t change.” (A2)
Theme 4: perceived benefits of MI for patient communication and clinical practice Final-year medical students described multiple benefits of applying MI in routine consultations, particularly in improving communication and strengthening the doctor-patient relationship. Students perceived MI as a valuable approach for establishing shared goals and involving patients in decision-making, rather than relying on 1-way directive advice (B3 and A2). This collaborative communication style helped patients feel listened to and respected, which in turn increased trust and engagement during consultations (B2 and A3). Many students reported that using MI enhanced patients’ motivation and willingness to participate in their own care. They observed that when patients were encouraged to express their concerns and motivations, they became more confident and proactive in managing their health conditions (B4 and C3). Students noted that this approach was particularly useful in managing chronic diseases such as diabetes, where long-term behavior change is essential for effective care (B5). Students also highlighted that MI helped them better understand patients’ perspectives, emotions, and barriers to behavioral change, thereby fostering greater empathy in clinical interactions (C3 and A1). Several participants emphasized that MI aligns with patient-centered and ethical communication, helping to reduce misunderstandings and potentially decrease the likelihood of patient dissatisfaction or complaints (C1). In addition to benefits for patient care, students reported that using MI positively influenced their own professional development. They described feeling more confident and satisfied when consultations became more collaborative and when patients responded positively to their communication approach (A1 and C3). Some students also noted that MI improved their broader communication skills, including active listening, reflective responses, and awareness of personal biases during clinical encounters (B5 and C2). Finally, several participants suggested that MI may contribute to more efficient long-term care. They perceived that patients who felt motivated and supported were more likely to engage in self-management, which could reduce repeated consultations for the same behavioral issues. For many students, practicing MI also reinforced their sense of professional identity as a physician who communicates with empathy, respect, and patient-centered care (A1, A2, and B2).	“This was the first thing that interested me—how to speak. It helps guide me a lot when talking with patients. I think it’s very important.” (B1) “This is a skill every doctor should have. Before, I would just tell patients what to do without exploring how difficult behavioral change can be or how much time it takes to change. Now I use a patient-centered approach, finding a shared middle ground together.” (B2) “MI is important. Giving advice is necessary, but advice alone is a one-way communication and then it ends. MI, on the other hand, allows patients to make their own decisions. If we do it well, good outcomes will follow—like quitting smoking and preventing disease. With better clinical practice, the results we aim for will also improve.” (A2) “We don’t force the patient to do anything — we plan together and see things from their perspective. It’s better than just telling the patient what to do. It helps us understand the ways that lead to change. Even though we don’t follow up with the patient afterward, during the interaction we can feel that the patient understands and has goals for change. It creates a good relationship with the patient.” (A3) “MI is important because it helps us find a shared goal. If the doctor doesn’t do this, the patient may not want to make any changes—it feels as if someone is just ordering them, so they don’t want to follow. MI helps patients want to improve themselves and want to collaborate with our guidance. The benefit for us is that it helps us understand patients better. It’s not just about applying a theory and expecting results to happen automatically.” (B3) “MI is important. It’s clear that patients become more cooperative. It’s evident that MI truly helps motivate patients, and it helps us understand them better — we gain more insight into their background and situation. Patients are more willing to cooperate as a result.” (B4) “Using MI in the care of patients with diabetes makes management easier, if it is used correctly and fluently.” (B5) “MI is very important. Behavior is something that stays with patients for long time. What happen is the patient is often not understood by the doctor because we’ve never asked about their thoughts and feelings—we still don’t really know each other. MI provides a natural way for us to do this. It gives us a structure to understand the patient. Behavior is hard to change. MI helps us reach the patient’s feelings and talk about their attitudes, which is extremely helpful.” (C3) “It benefits us by helping us empathize with patients and listen to them more attentively. Sometimes we don’t feel like listening to patients, even when what they say affects their treatment. It reminds us not to let our own bias get in the way, and to act professionally. It also helps us observe the patient’s gestures and behavior.” (B5) “MI is important, yet it’s something no one has ever brought up. MI is a part of medical ethics—listening without judgment and applying the principles of patient-centered care. We didn’t recognize its importance before or realize what proper ethical principles really entail—we just practiced without knowing their significance.” (C1) “The benefit for myself is that I feel, in my heart, like a good doctor. The patient is happy, and they leave the consultation with a sense of satisfaction.” (A1) “It helps patients open up to us more, as if we were talking with a family member. When we encourage them and give positive support, that positivity comes back to us. The patient is happy, and we are happy — we receive positive energy. It makes us appreciate the value of caring for patients even more.” (C3) “The benefit for ourselves is improved communication skills, which help not only with patients but in general as well.” (C2) “The outpatient setting is very important. If there is proper guidance on how to speak with patients, it helps them feel comfortable and satisfied when they listen, and they become more cooperative. That’s exactly what MI does.” (A1) “Doctor–patient relationship becomes more open, allowing patients to trust the doctor and feel understood. It gives them the opportunity to think for themselves, and they feel more confident and trusting. As a result, our work improves and becomes lighter—our workload is reduced because patients understand how to take care of themselves.” (A2)
Theme 5: development of confidence in applying MI After completing the MI course and practicing its techniques during clinical rotations, most final-year medical students reported increased confidence in communicating with patients and supporting behavioral change (B1, B3, and B4). Several students noted a shift from feeling uncertain or hesitant in offering guidance before the course to becoming more comfortable and proactive in conversations involving lifestyle changes or complex emotions (B4 and C3). Students described how MI gave them a clearer framework for patient interaction, helping them move beyond simply giving advice to learning how to guide patients through reflective discussions and motivate change (A3 and B5). A few emphasized that increased confidence was also tied to stronger medical knowledge, which enabled more fluent and effective use of MI in practice (A1). Although confidence was not immediate for everyone, many students shared that through repeated use—both in clinical encounters and informal settings—they became more adept at integrating MI naturally into their consultations (A2 and B5).	“More confident in giving advice to patients, more willing to speak up, and becoming more familiar with it.” (B1) “More confident, and during OPD rotations I use MI more often.” (B3) “More confident now. Before the course, I didn’t dare to give advice to patients.” (B4) “I am more confident in helping patients change their behavior. At first, I didn’t know there was a principle behind it, I would just give advice or tell them what to do. In the future, I will use MI in situations where it is appropriate, but there are still limitations due to the high number of patients coming to the OPD.” (B5) “To be confident in speaking and using MI, we must first be confident in our medical knowledge. Once we are confident in our knowledge, we will feel more confident during patient consultations, and the more we speak, the more fluent and confident we become in using MI.” (A1) “More confident immediately after the course, but not at the highest level yet. After practicing for a while following the course, I became much more confident. I tried using it on my own and no longer needed additional training.” (A2) “Before learning MI, everything was scattered and without direction. I didn’t know what to do and had no framework. Now, I feel more confident.” (C3) “There is now a clearer starting framework. Before, I would give advice based on the knowledge I had — 1, 2, 3, 4 — without giving patients the chance to talk about why they couldn’t follow it. After learning MI, I understand patients more, can help motivate them, and can apply a more effective approach to giving advice.” (A3)
Theme 6: students’ perspectives on when MI should be taught in the curriculum Most final-year medical students agreed that year 5 is the most appropriate time to introduce MI in the curriculum, as it aligns with outpatient clinical exposure and provides enough time to practice before entering the externship year (B1, B2, and B4). Students noted that by year 5, they have sufficient medical knowledge and experience to make MI training meaningful and applicable, especially during rotations involving real patient care and behavioral counseling (A3 and B3). A few students recommended introducing MI theory earlier, in years 3 or 4, followed by more practical reinforcement in years 5 and 6 to help develop fluency over time (C1 and A2). Some felt that teaching MI too late—such as near the end of year 6—would limit the opportunity to apply and refine skills in varied clinical settings (B2 and A2). However, a small number suggested that year 6 is ideal, viewing it as the stage when clinical confidence matures enough to fully integrate MI into patient care (B5 and C3). Overall, the majority favored a staggered approach, with early theoretical exposure followed by increasing depth and practice in years 5 and 6.	“I would like to learn it before entering year 6, so that I can practice more and apply it across different block rotations. It could be early in year 5 or during the family medicine rotation in year 5. Since in year 5 we have OPD sessions, while in year 4 we mostly focus on history taking and physical examination, learning it in year 5 would be better. Then, by the time we reach year 6, when we are more involved in the patient management, we would be able to use it more fluently.” (B1) “In years 5 and 6, there are more opportunities to explore patient care. In year 5, there is the OPD Ambulatory Medicine course. If MI is taught in year 5 and then applied in Ambulatory Care in Primary Care during year 6, that would be ideal. However, in year 6, if a student takes the block toward the end of the year, they would learn the techniques late and have less time to practice. Therefore, learning MI in year 5 would be better.” (B2) “Years 4‐5 are okay because that’s when we start working in the outpatient department. In year 4, we have a rotation in community hospitals, and I heard that the nurses there use MI with diabetic patients. So if we could learn it in year 4, that would be good. Then in years 5‐6, we would be able to apply it in real practice.” (B3) “Learning it in year 5 would be good because we have outpatient rotations and can apply it. By year 6, we’d have a solid enough understanding to use it directly in clinical practice.” (B4) “I think teaching it during the extern year (year 6) is suitable because students already have confidence in patient care. But if it’s taught before the extern year, students may not be ready yet.” (B5) “MI should start being taught theoretically in years 3‐4. After learning the theory, students can then observe patients, and later receive reinforcement teaching again. In year 4, the focus should be on engaging and focusing, because that year is mainly about history taking and physical examination. In years 5‐6, the teaching should shift to evoking and planning, because students begin learning about treatment and management. This would help students understand MI more deeply. Those who already enjoy talking with patients benefit a lot when they learn MI, but those who are more academically focused, not like to listen or talk may not use it as much.” (C1) “In year 4, it can be practiced during the family medicine rotation. If students start practicing in year 4 and continue in years 5‐6, their skills will become more complete. If it is taught near the end of the extern year, they may not have many opportunities to apply it in different wards.” (A2) “Year 6, because that’s when we get to use it in real practice. In year 4, during OPD rotations, it’s difficult to apply because most of the learning is through lectures and observing instructors. If we had to do it ourselves, it would be hard to apply, since the focus in that year is mainly on history taking and physical examination.” (C3) “Years 5‐6 are suitable because by the time we reach the stage of giving advice, we already have the necessary knowledge and understand the importance of advising patients. If it is taught too early, we won’t have enough information or knowledge to really help patients. If it is taught too early, we will forget it, won’t see its importance, and won’t be focused. In years 5‐6, students are more likely to want to use it.” (A3)
Theme 7: preferred educational approaches for learning MI Final-year medical students emphasized the importance of combining lectures with hands-on practice when teaching MI. While onsite lectures and web-based modules were considered helpful for providing foundational knowledge (B1, B3, and B4), most students stressed that practice-oriented learning—especially through role-play, video demonstrations, and feedback or coaching sessions—is essential for building confidence and applying MI effectively in real clinical settings (B2, C1, and C2). Several students recommended a flipped classroom model, where theoretical content is delivered online before in-person sessions focused on skill application and reflection (B3 and B4). They also suggested that extended or repeated practice sessions, including opportunities to rehearse full MI processes and observe expert demonstrations (eg, videos or live modeling), would help address initial confusion—particularly around more complex components such as evoking and planning (A3 and B5). Overall, students viewed a combined educational approach—with early theoretical input, case-based role-play, instructor feedback, and opportunities for ongoing practice—as key to mastering MI during undergraduate medical training.	“Onsite lectures are good, but there is still not enough practice. When applying it with patients, there is still a lack of feedback. I would like there to be a practice session with feedback or coaching. Video demonstrations are useful and could be added to the web-based course, or a workshop could be organized for students or physicians who are interested, perhaps as an elective.” (B1) “I was impressed by the session where we practiced brief MI. I was able to try applying it with patients myself. The lecture is important, but I would like to have hands-on practice right away. The web-based format is already detailed and complete. We could watch the online clips before, during, or after trying it with patients. For the theory part, it should be summarized briefly and concisely, and then we should move straight to practice.” (B2) “Lecture+ web-based learning is already good. But if there were a wrap-up session, it would be even better, because in the classroom I couldn’t keep up. If we could study the web-based content first and have some time to digest it, we would understand the in-class session better, since this is a new topic for us. Web-based learning is helpful because I can’t always follow everything during the live class. Role-play and videos would also be useful because they make it easier to apply MI and help build confidence in using it.” (B3) “I prefer learning onsite and then reviewing it again online. Another option is to have students learn the theory on their own through the web-based course, and then the instructor can give a brief summary in class and let students do role-play.” (B4) “The teaching format is appropriate as it is. However, the session where we practiced brief MI was too short. The session should be extended, and we should also try practicing the full MI process. I would like to add a video demonstration because the current case studies are only on paper. After this, reviewing the principles doesn’t require additional courses—I think we can continue practicing on our own.” (B5) “What I remember most from the course is how to speak well with patients. Even though it’s been a long time, I still remember it clearly because I saw the instructor demonstrate it during the OPD rotation, especially how to approach difficult patients. The instructor was a good role model, and seeing the correct practice has stayed with me to this day.” (A1) “Lectures alone may not be enough—there should be more practice included. Perhaps add role-play demonstrations for difficult patients, with role-play sessions that classmates can observe. Coaching is challenging, but it would be very helpful if available. The content is sufficient, but what’s missing is how to apply it—how to practice correctly and in what way. Skills should be refreshed regularly through small sessions, such as annual courses or workshops, so that when we apply them in practice, we have a solid foundation to rely on.” (C1) “Organize group role-plays in front of the class and have the instructor provide feedback and advice. For physicians who want to use it, the training is sufficient, but continuous practice is necessary.” (C2) “I could follow about 50% of the lecture. When practicing, I didn’t really understand what the evoking process meant or how to use it. I was still confused when trying it, but once I understood the concept, I felt that if we practiced with roleplay first and then had a follow-up lecture, it would be better. Learning from video demonstrations is difficult if we don’t immediately encounter real cases. Maybe we could divide into groups for roleplay, then go back and watch the videos, and the next day do hands-on practice—that might work better. More time should be given for learning and self-practice, with more opportunities to practice.” (A3)

a MI: motivational interviewing.

b NCD: noncommunicable diseases.

## Discussion

### Principal Findings

This mixed methods evaluation examined a combined MI training program for final-year medical students. Quantitative findings showed higher MI knowledge scores and greater self-reported confidence after participation in the training program. In addition, most students reported applying elements of MI during clinical encounters, primarily in brief interactions related to counseling patients with chronic diseases.

Qualitative findings provided further insight into students’ learning experiences. Participants described increased awareness of patient-centered communication strategies, including reflective listening, open-ended questioning, and affirmations. However, students also reported challenges in applying more advanced MI processes, such as evoking and planning, due to time constraints, clinical workload, and limited opportunities for repeated practice. Together, these findings suggest that the combined learning approach was associated with improvements in knowledge and perceived communication confidence, while also highlighting practical challenges in implementing MI during routine clinical encounters.

It is important to note that these findings reflect self-reported learning outcomes and perceptions of MI use, rather than objectively measured MI competency or verified patient behavior change.

### Comparison With Previous Studies

Our findings are broadly consistent with previous studies suggesting that brief MI training can increase learners’ knowledge and confidence in behavior-change communication [18,19,35,36]. Similar to prior research, students in this study reported feeling most comfortable using the early MI processes of engaging and focusing, while evoking and planning were more difficult to apply without extended practice or supervision. Previous research has also suggested that blended learning approaches combining online instruction with interactive teaching may enhance learner engagement and flexibility in medical education [28].

However, it is important to note that many studies evaluating MI education rely primarily on self-reported outcomes, and evidence regarding objective skill acquisition remains limited. Therefore, while this study contributes additional evidence from a Thai undergraduate medical education context, further research using objective assessment tools and controlled study designs is needed to better evaluate the effectiveness of MI training programs for undergraduate medical students.

Consistent with qualitative findings, students perceived limited consultation time as a barrier to applying MI. These perceptions are consistent with previous reports from trainees who are newly learning MI techniques and may require additional time to formulate reflective responses and guide behavior-change conversations [27]. However, evidence from the broader MI literature suggests that when clinicians become more proficient in MI, the approach can be incorporated efficiently into routine clinical consultations without substantially increasing encounter time [9,37]. Some studies have even reported that effective MI communication may improve the efficiency of patient interactions by helping clinicians focus more quickly on patients’ motivations and priorities for behavior change [9,38]. Therefore, the time-related challenges reported by students in the present study may reflect their early stage of learning rather than an inherent limitation of MI itself. As students gain more experience and practice integrating MI skills into clinical encounters, the perceived time burden may decrease.

### Strengths and Weaknesses

A major strength of this study lies in its mixed methods design, which allows integration of quantitative findings with qualitative insights to provide a broader understanding of students’ learning experiences. The inclusion of an entire cohort of final-year medical students reduced the selection bias and enhanced representativeness. The use of validated instruments and expert review increased internal validity.

Several limitations should be considered when interpreting the findings of this study. First, this study used a single-group before-and-after design without a control group, which prevents causal attribution of observed changes to the educational intervention. Improvements in knowledge or confidence may also reflect concurrent clinical learning experiences, maturation effects, or testing effects during the academic year.

Second, several outcomes, including students’ reported application of MI in clinical encounters, were based on self-reported measures, which may be subject to recall bias or social desirability bias. These measures, therefore, reflect students’ perceptions rather than objectively verified MI competence. In addition, the qualitative interviews were relatively brief (approximately 15‐20 minutes) due to scheduling constraints during students’ clinical rotations, which may have limited the depth of exploration of participants’ experiences.

Third, the duration of the intervention was relatively brief (approximately 5 hours of combined learning), which may be sufficient for introducing foundational MI concepts but is unlikely to support mastery of more advanced MI skills without continued practice, feedback, and supervision [10,39].

The relatively low completion rate of the web-based module may also reflect challenges related to implementation fidelity, as not all students were fully engaged with the online component of the intervention. However, the web-based course was designed as a supplementary resource to support students who wished to review or reinforce knowledge delivered during the onsite sessions. Participation in the web-based module was optional, and therefore variations in uptake among students were expected. As this study was not originally designed to compare outcomes between students who completed the web-based module and those who did not, subgroup analyses were not conducted. Future studies may explore whether different levels of engagement with online learning components influence educational outcomes.

Finally, this study was conducted at a single medical school, and therefore, the findings may not be generalizable to other educational settings or health care systems.

### Implications and Future Study

The findings of this study offer practical implications for undergraduate medical education. A combined learning structure that integrates a short workshop with a self-paced web-based module may provide a flexible approach for introducing MI concepts within a busy curriculum, although engagement with the online component varied among students. The web-based component provides flexibility, while the in-person workshop facilitates experiential learning and peer interaction.

Students’ preference for learning MI during the fifth year supports the alignment of MI training with clinical exposure to maximize relevance and skill transfer. A spiral curriculum, introducing MI principles during preclinical years and reinforcing practical skills during clinical rotations, could further strengthen integration.

Addressing barriers such as workload and limited practice time will require institutional support, including formative feedback, supervision, and longitudinal reinforcement. Embedding MI within broader communication skills training and assessment frameworks may enhance sustainability.

Future research should incorporate objective measures of MI competency, such as validated behavioral coding systems or structured communication assessments, to more accurately evaluate skill acquisition. Longitudinal studies are also needed to examine the retention of MI skills and their potential impact on patient outcomes over time. In addition, multicenter and cross-cultural research in low- and middle-income countries would help determine how combined MI educational approaches can be adapted to different health care systems and educational environments.

Overall, the findings suggest that the intervention was feasible within an undergraduate medical curriculum and was associated with higher knowledge scores and increased perceived confidence among participating students, while qualitative findings illustrated how students perceived and attempted to apply MI principles during clinical training.

### Conclusions

In summary, this study evaluated a combined educational approach comprising a brief workshop and a web-based learning module to introduce MI to final-year medical students. The findings suggest that the training was associated with improvements in students’ MI knowledge and self-reported confidence in behavior-change communication. Students also reported applying elements of MI during clinical encounters, particularly when counseling patients with chronic diseases.

While these findings indicate that combined MI education is a feasible approach for introducing communication skills within undergraduate medical curricula, the results should be interpreted cautiously due to the absence of a control group and the reliance on self-reported outcomes. Further research using controlled study designs, objective assessment tools, and longitudinal follow-up is needed to better evaluate the impact of MI training on clinical competency and patient outcomes.

## Abbreviations

MI: motivational interviewing
